# Utilization of a precision medicine genetic and psychosocial approach in outcome assessment of bariatric weight loss surgery: a narrative review

**DOI:** 10.3389/fpubh.2025.1516122

**Published:** 2025-05-01

**Authors:** Colin Hanna, Fiona Comstock, Shtakshe Chatrath, Alan Posner, John Butsch, Kenneth Blum, Mark S. Gold, Lesley Georger, Lucy D. Mastrandrea, Teresa Quattrin, Panayotis K. Thanos

**Affiliations:** ^1^Behavioral Neuropharmacology and Neuroimaging Laboratory on Addictions, Department of Pharmacology and Toxicology, Clinical Research Institute on Addictions, Jacobs School of Medicine and Biosciences, University at Buffalo, Buffalo, NY, United States; ^2^Department of Surgery, Jacobs School of Medicine and Biomedical Sciences, University at Buffalo, Buffalo, NY, United States; ^3^Division of Addiction Research & Education, Center for Exercise Sports & Global Mental Health, Western University Health Sciences, Pomona, CA, United States; ^4^Department of Molecular Biology, Adelson School of Medicine, Ariel University, Ariel, Israel; ^5^Department of Psychiatry, Washington University in St. Louis, St. Louis, MO, United States; ^6^Department of Natural Sciences and Mathematics, D'Youville University, Buffalo, NY, United States; ^7^UBMD Pediatrics Division of Endocrinology/Diabetes, Buffalo, NY, United States

**Keywords:** obesity, drug abuse, addiction, reward deficiency, hypodomanergia, gastric bypass, vertical sleeve gastrectomy, personalized medicine

## Abstract

The obesity epidemic has become a global public health issue, impacting more than one billion people worldwide. 9% of the US population, or 28.8 million Americans will have an eating disorder in their lifetime. In fact, global eating disorder prevalence increased from 3.5% to 7.8% between 2000 and 2018. In spite of the fact that less than 6% of people with an eating disorder are medically underweight, it is indeed an important factor when considering issues related to obesity. This public health problem is often described as being caused by various genetic and psychosocial factors. One of the most effective strategies for treating morbid obesity and achieving significant weight loss is bariatric surgery. Recent focus on precision medicine approaches has expanded into bariatric surgery in an effort to better understand and achieve improved outcomes and reduce risk for post-operative weight regain and addiction transfers during the recovery process. Addiction transfers, including substance and non-substance addictions, are well established concerns for post-bariatric patients. This review details the genetic, molecular and psychosocial factors that can be utilized to inform and guide personalized treatment. Additionally, this review details some of the molecular mechanisms including dysregulation of catecholamine signaling as well as other neurotransmitter systems relevant to help further understand recovery science.

## 1 Introduction

Obesity is a growing epidemic affecting more adults each year. In 2016, 1.5 billion adults were impacted by obesity worldwide (1). This problem is projected to persist by the year 2030, with an approximated 1.35 billion overweight individuals followed by 573 million obese adults (1, 2, 159). Due to varying patterns in fat and body composition, the geographic concentration of obese individuals is greater for much of Asia, Latin America, the Middle East, and Africa.

Additionally, obesity is a risk factor for some of the most prevalent adult diseases. It was found that an increased 40 billion dollars in medical spending is required annually for obesity-related health problems (2). Insulin resistance, dyslipidemia, and high blood pressure are common comorbidities in obese patients, and there is ongoing research on possible genetic ties to these diagnoses (3). Additional comorbidities that have been associated with obesity include cardiovascular disease, some cancers, kidney disease, obstructive sleep apnea, gout, osteoarthritis, and hepatobiliary disease, many of which can shorten lifespan (4, 5). Decreasing worldwide prevalence of obesity would thus improve overall health.

While there are various FDA approved anti-obesity medications available to the public, some of these medications can present adverse effects to patients and can be costly. Bariatric surgery is an effective means of weight loss for individuals who have been unsuccessful with traditional weight loss methods (6, 7). One common failed method of weight loss includes the implementation of a restrictive diet, such as a diet low in carbohydrates. There is no significant advantage of low carbohydrate diets compared to traditional nutritionally balanced, energy restricted diets, and low carbohydrate intake can lead to further complications such as heart arrhythmias, kidney damage, osteoporosis, increased cancer risk, and more (8). Some have concluded that in comparison to dietary methods, exercise, pharmacotherapy and behavioral therapy, bariatric surgery is the most effective means of weight loss in obese patients (9).

In the context of severe obesity, both bariatric surgery and GLP-1 receptor agonists (GLPs) have proven to be effective treatments. However, the decision on which treatment is appropriate for a particular individual depends on various factors, including the severity of obesity, presence of comorbidities, previous weight loss attempts, and patient preferences (see Table 1). The choice between bariatric surgery and GLP-1 receptor agonists for the treatment of severe obesity should be personalized based on individual patient characteristics, preferences, and clinical circumstances. Both options can lead to significant weight loss and improvement in obesity-related comorbidities, but they come with different risks and benefits that need to be carefully considered (6, 10–12)

**Table 1:** A summary of the considerations of treatment modalities for obesity.

	**Bariatric Surgery**	**GLP-1 Agonists**	**References**
Candidates for treatment	• Severe Obesity (BMI ≥40 kg/m^2^) • Moderate Obesity with Comorbidities (BMI 35-39.9 kg/m^2^), such as Type 2 Diabetes Mellitus, Hypertension, Obstructive Sleep Apnea • Failure of non-surgical treatments, such as diet, exercise, and pharmacotherapy • Psychological readiness to undergo surgery and make necessary lifestyle changes postoperatively • Absence of contraindications, such as certain psychological conditions or substance abuse	• Moderate to Severe Obesity (BMI ≥30 kg/m^2^ or ≥27 kg/m^2^ with Comorbidities) • Preference for non-surgical treatment, due to either preference or contraindications • Adjunct to lifestyle modifications for those willing to change lifestyle alongside pharmacotherapy • Good response to GLP-1 Agonists	(142, 143)
Benefits	• Significant and sustained weight loss • Improvement or resolution of comorbid conditions • Enhanced quality of life • Potential reduction in mortality	• Significant weight loss, though typically less than that achieved with bariatric surgery • Improvement in glycemic control, particularly beneficial for patients with type 2 diabetes • Non-invasive option with fewer immediate risks compared to surgery	(142, 144)
Risks	• Surgical risks such as infection, bleeding, and anesthesia complications • Long-term risks like nutritional deficiencies and the need for lifelong follow-up	• Gastrointestinal side effects such as nausea, vomiting, and diarrhea • Potential for more serious adverse effects such as pancreatitis • Long-term safety profile still under study	(145)

Regarding bariatric surgery, the two most common types of procedures include laparoscopic sleeve gastrectomy (LSG) and Roux-en-Y gastric bypass (RYGB). One study reported that after 7 years, the success rates 47% weight and 55% weight loss respectively, and both surgeries improved quality of life of the patients (13). While bariatric surgery has proven to improve the lives of many individuals suffering from obesity, there are also potential risks that bariatric surgery may pose. These risks include post-operative weight regain as well as post-surgical complications including adhesions, intestinal leak, etc. In addition, several behavioral risks can be revealed after bariatric surgery. In fact, it has been found that many substance and non-substance behavioral addictions tend to increase after obesity operations (14). These risks can be assessed proactively by administering baseline psychological screenings to identify traits that patients experience before surgery. Obtaining this data can help inform psychological treatment post-surgery (6, 10–12).

**Figure 1:**
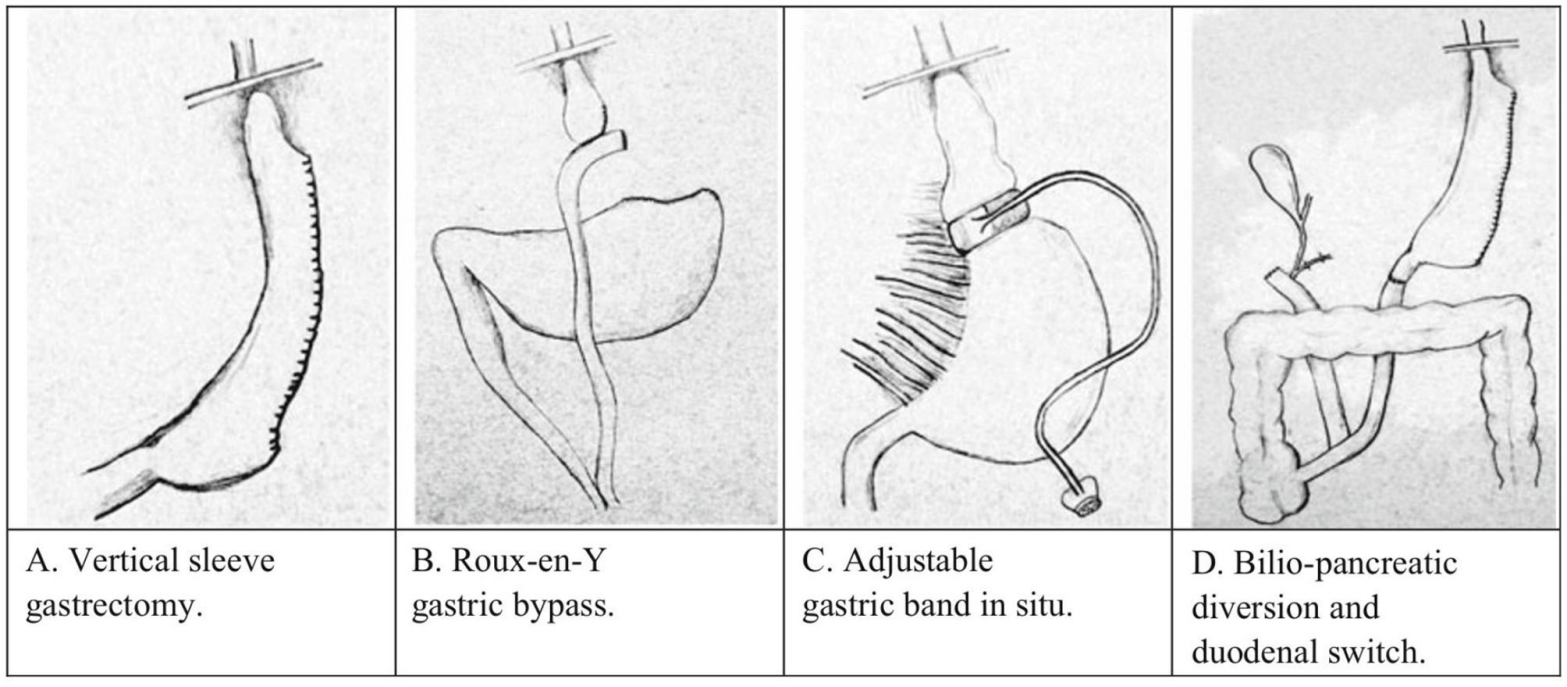
Types of bariatric surgery in humans as adopted from (141). **(A)** Vertical sleeve gastrectomy, **(B)** Roux-en-Y gastric bypass, **(C)** adjustable gastric band, and **(D)** bilio-pancreatic diversion and duodenal switch.

This review is a thorough description of the facets of obesity and bariatric surgery (Figure 1) that crossover to addiction and reward processes. This narrative review was conducted by searching PubMed electronic databases utilizing the search terms “Obesity” “addiction”, “Dopamine”, “hypodomanergia”, “gastric bypass”, “vertical sleeve gastrectomy”, and others. In addition, genes associated with reward processing were searched in combination with variables related to obesity.

## 2 Obesity and bariatric surgery (history of bariatric surgery, types, and outcomes)

The first bariatric surgery, deemed “jejuno-ileal bypass” was performed in 1954 (Kremen, Linner, and Nelson 15). Though this form of surgery introduced many risks, such as dehydration and diarrhea (15), patients with high cholesterol achieved normalized lipid levels, and resolution of diabetes/prediabetes, reduced hypertension and sleep apnea (16–23). The first form of modern bariatric surgery was performed in 1966, after it was noted that cancer patients who underwent sub-total gastrectomy lost large amounts of weight. Initially, the procedure consisted of a horizontal gastric transection with a loop ileostomy, but it was later optimized to smaller gastric pouches and stoma sizes (24). The “Roux-en-Y” loop, which diverts bile from the stomach and the esophagus, decreases bile reflux (25). This form of gastric bypass decreased the risk for diarrhea, dehydration, kidney stones and gallstones (15). The Fobi-Capella banded gastric bypass is a method designed to boost weight loss by using a ring to constrain gastric pouch enlargement and curb weight regain (26).

Performing laparoscopic RYGB presents significant technical challenges, characterized by a steep learning curve and the potential for leaks at two points of anastomosis (15). Given the technical hurdles in laparoscopic surgery for patients with Class 3 obesity, Gagner (27) proposed a staged procedure from the original Scopinaro type biliopancreatic diversion (28), initiating the process with a vertical gastrectomy (sleeve) followed by the duodenal switch (27). This stepwise approach, including sleeve gastrectomy as the first stage, had been advocated due to its effectiveness, as evidenced by significant weight loss outcomes (56%) (15).

A study by Nasser et al. detailed some of the different rationales behind performing each type of bariatric surgery (29). Sleeve gastrectomy is currently the most performed bariatric surgery. Generally, the decision to perform RYGB is based on higher BMIs and obesity comorbidities, such as diabetes mellitus, gastroesophageal reflux disease, BMI ≥ 50 kg/m^2^, obstructive sleep apnea, hypertension, hyperlipidemia, and American Society of Anasthesisiologists (ASA) class > 3. The ASA classification measures a patient's preoperative risk on a scale of 1 to 5 based upon physiological status and comorbid conditions (29–31). Additionally, it was found that sleeve gastrectomy was performed more often in patients who were deemed “high risk”, which included a history of smoking, steroid use, kidney disease, and chronic obstructed pulmonary disease (29). Additionally, although all bariatric surgery types pose a risk for addiction transfer and alcohol misuse, RYGB is generally understood to pose a greater risk for post-surgical alcohol consumption compared to other types of surgery (32–35).

A longitudinal study by Salminen et al. compared weight-loss outcomes between the two common procedures in 240 patients seven years after surgery at 5 years, 7 years, and 10 years (13, 36). After 5 years, The mean percentage of excess weight loss at 5 years was roughly 49% after sleeve gastrectomy and approximately 57% after gastric bypass surgery. The difference between the two groups was around 8.2 percentage units, favoring gastric bypass. These findings did not indicate equivalence between the two procedures (36). After 7 years, following sleeve gastrectomy, the mean percentage of excess weight loss (%EWL) was approximately 47% (CI 43–50%). After RYGB, the %EWL was roughly 55% (CI52%−59%). The difference between the two procedures was around 8.7 percentage units (CI 3.5–13.9 percentage units), favoring RYGB (13).

At 10 years, the median excess weight loss (%EWL) was 43.5% following LSG and 50.7% following RYGB. On average, %EWL differed between the procedures; RYGB had an 8.4% higher %EWL. There were no statistically significant differences in type 2 diabetes remission, dyslipidemia, or obstructive sleep apnea post-LSG and RYGB. However, hypertension remission was better after RYGB (8% vs. 24%; *P* = 0.04). Esophagitis occurred more frequently after LSG (31% vs. 7%; *P* < 0.001) (37).

A clinical trial by Peterli, compared the outcomes between sleeve gastrectomy and gastric bypass 5-years after the procedure (38), finding that Gastric reflux showed a higher rate of remission following RYGB (60.4%) compared to sleeve gastrectomy (25%). Conversely, gastric reflux worsened, as measured by symptoms or escalation of medical therapy, more frequently after LSG (31.8%) than after RYGB (6.3%). Reoperations or interventions were needed for 16 out of 101 patients (15.8%) after sleeve gastrectomy and 23 out of 104 patients (22.1%) after RYGB. There was no significant difference in weight loss between the two procedures in their cohort (38).

Gut microbiotia is a growing area of research in the field of bariatric surgery. Obesity leads to decreased gut microbiota diversity and increased micronutrient deficiencies, and bariatric surgery alters gut microbiota composition and impacts the synthesis of vitamins like riboflavin, folate, B12, and vitamin K2 (39). Gutiérrez-Repiso analyzed the gut microbiota of 76 patients undergoing sleeve gastrectomy, classifying them into responder and nonresponder groups based on weight loss after one year (40). It was found that the responder group had a distinct gut microbiota composition, with a higher Prevotella-to-Bacteroides ratio compared to the nonresponder group before surgery, which could potentially predict weight loss outcomes. After surgery, the responder group showed an increase in microbiota linked to beneficial metabolic effects, suggesting that preoperative gut microbiota may influence bariatric surgery success (40).

Additionally, further intervention must be considered following bariatric surgery to ensure desirable results. Currently, it has been determined that within 5 years of bariatric surgery, 50% of patients experience weight regain and comorbidity relapse. Exercise is recommended following bariatric surgery to ensure optimal outcomes; however, a systematic review of 28 studies suggests that exercise intervention is poorly conducted in patients post-bariatric surgery. It is essential that this deviation be resolved as resistance and aerobic training support healthy weight, bone and cardiometabolic health, as well as aerobic capacity following bariatric surgery (41) (see Table 2).

**Table 2:** An overview of documented outcomes comparing the two most common types of bariatric surgery: Roux-en-Y gastric bypass and sleeve gastrectomy.

	**Roux-en-Y gastric bypass**	**Sleeve gastrectomy**	**Reference**
Performed on patients for the following reasons:	° High BMI ° Comorbidities: diabetes, gastroesophegal reflux, sleep apnea, hypertension, hyperlipidenea	° High-risk patients (smokers, steroid users) ° Kidney disease ° Obstructed pulmonary disease	(29)
Weight loss	57% EWL (5 years) 68.3% EWL (5 years) 55% EWL (7 Years) 50.7% EWL (10 Years)	49% EWL (5 Years) 61.1% EWL (5 years) 47% EWL (7 Years) 43.5% EWL (10 years)	(13, 36, 37)
Gastric reflux Remission	60.4%	25%	(38)
Gastric reflux worsening	6.3%	31.8%	(38)
Reoperations	22.1%	15.8%	(38)
Diabetes remission	47%	33%	(146)
A. Alcohol use problems B. *De novo* alcohol-related diagnosis C. Unhealthy alcohol use	A. HR: 1.86 B. AHR: 1.51 C. 9.2%	A. HR: 1.35 B. AHR = 0.77 C. 7.9%	A. (33) B. (34) C. (35)

Documented outcomes include: Choice between the two surgeries, weight loss outcomes, gastric reflux outcomes, rate of reoperations, diabetes remission, and alcohol misuse. EWL, Expected Weight Loss; HR, Heart rate.

## 3 Risk of addiction transfer

Reward Deficiency Syndrome (RDS) serves to measure the role of epigenetics and genetics in compulsive behaviors such as gambling, binge eating, alcohol and drug abuse (5). This offers a genetic descriptive of Pre-Addiction, or simply put, a predisposition to addiction behaviors (42). Further, the Genetic Addiction Risk Severity (GARS) assay is used to detect common polymorphisms related to RDS (43). A few of these polymorphisms include DRD2, DRD3, DRD4, DAT1, COMT, OPRM1, and 5HTT polymorphisms surgery, a few of which being alcohol addiction, drug addiction, and gambling (44). Alcoholism, specifically, frequently develops in patients post-bariatric surgery (45). It has been found that within 5 years of undergoing RYGB surgery, approximately 20% of patients developed alcohol use disorder (AUD) as their symptoms of food overconsumption decreased with post-surgical weight loss (45) The literature is conflicting on whether the choice of bariatric surgery procedures increases the risk of AUD. Although all bariatric surgery types pose a risk for addiction transfer and alcohol misuse, RYGB bypass is generally understood to pose a greater risk for post-surgical alcohol consumption compared to other types of surgery such as laparoscopic adjustable gastric banding (32–35, 46). Conversely, further research has suggested that there is no significant difference in the incidence of AUD following RYGB bypass compared to LSG procedures—which currently accounts for over half of all primary bariatric surgeries (47). While addiction risk does pose a serious threat to patients, bariatric surgery is still an attractive option for patients suffering from obesity as the mortality rates of obese patients post-bariatric surgery have significantly decreased (48–51).

There is, however, conflicting evidence on addiction transfer in bariatric populations (52, 53). Dickhut et al. conducted a study in 49 patients undergoing bariatric surgery (52). In this study, various measures of impulsive and compulsive behaviors were collected including those on alcohol intake, internet use, gambling, shopping, and sex addiction. In this study, no new addiction symptoms emerged and many of these scores significantly decreased at 1-year follow ups (52). Another study found that while both sleeve gastrectomy and gastric bypass significantly reduced food addiction in obesity patients, neither procedure led to cross-addiction, with no significant differences between the two surgical methods (53).

There are reports that psychiatric risks including substance abuse, self-harm and even suicide are matters of concern alongside post-surgical addiction transfer, with the greatest risk occurring 1–3 years post-surgery (54). In fact, it was found that the endorsement of substance misuse was related to a lower percentage of post-surgical weight-loss (55). Post bariatric substance misuse was also associated with a family history of substance misuse and residual psychological symptoms of food addiction, including nocturnal eating and selective hunger (55). One study conducted semi-structured interviews among 24 bariatric patients in substance abuse treatment programs (56). Three-quarters of patients recognized unresolved psychological issues, while over four-fifths pinpointed addiction transfer/substitution. Additionally, more than half observed quicker onset or heightened effects from substances, and nearly half noted increased accessibility of pain medications (56). Taylored psychotherapeutic techniques such as motivational interviewing and cognitive behavioral therapy can be utilized to promote health habits, including physical exercise and healthy eating (54, 57).

## 4 Genetics

The brain reward cascade includes the serotonergic, GABAergic, endorphinergic, cannibinergic, glutaminergic, cholinergic, and dopaminergic pathways. Specifically, the dopaminergic pathway is endpoint for reward in the brain. Imbalanced dopamine can bring about symptoms such as anhedonia, lack of motivation, and troubles coping with stress. Since psychoactive substances and addictive behaviors induce dopamine release, these behaviors are often seen in the use of individuals exhibiting this hypodopaminergic state (58). While RDS is an indicator for compulsive eating behaviors, the presence of these alleles also accounts for compulsive behaviors such as gambling and drug addiction. It has been identified that risk of addiction can be evaluated through the presence of various polymorphisms that play a role in compulsive behaviors (such as overeating) (59, 60), vulnerability to pain (58), and behavioral/conduct disorders (61). Specifically, there is a significant risk of alcohol use disorder in the presence of MAO, DRD1, DRD2, DRD3, DRD4, DAT1, COMT, OPRM1, GABABR3, and 5HTT polymorphisms (62). Alterations in these markers also applies to RDS which establishes a framework for these epigenetic behavioral expressions (63). The GARS test was developed by Dr. Kenneth Blum's research group and assesses 10 genes and 11 risk alleles that have been associated with substance and non-substance addictions (64). Specifically, the GARS test assesses an individual for RDS. RDS can be defined as a susceptibility to pain, addiction, and related behaviors. Various polymorphisms are assessed in the GARS such as DRD1, DRD2, DRD3, DRD4, MOA-A, COMT, DAT1, OPRM1, 5HTTLLR, and GABRA3 which are factors in the vulnerability of an individual to addiction and related compulsive disorders (59). Research on these polymorphisms has revealed a role in the body's pain mechanisms as well as a link between the OPRM1 gene and heroin abuse, the DRD2 gene and a high risk of heroin dependence, and the COMT gene linked to the response of opiates and enkephalins (65). Significant associations have also been found between the DRD3, DRD4, DAT1, COMT, OPRM1, and 5HTT genes and AUD (62). One study revealed that 77% of subjects known to have AUD contained the A1 allele of the D2 receptor gene, and 72% of participants without AUD did not have the A1 allele of the D2 receptor gene (66). One allele assessed for in the GARS assay, DRD2, has a Bayesian predictive value of 74% for detecting RDS behaviors. These various SNPs have significant effects on behaviors and addictions due to their role in brain pathways. Thus, testing for correlated alleles that alter these brain pathways, such as the DRD2 allele, can aid in the planning for adverse post-surgical addiction transfer. An overview of these genes and their associated addiction risks from documented clinical studies can be found in Table 3.

**Table 3:** An overview of the various addictive behaviors (substance and non-substance) associates with the SNPs as assessed by GARS.

**Substance/behavior of abuse**	**Associated gene**	**Gene function**	**Reference**
Heroin abuse and dependence	OPRM1, DRD2	Opioid Receptor Mu 1, Dopamine Receptor D2	(65, 147)
Alcohol use disorder	DRD2, DRD3, DRD4, DAT1, COMT, OPRM1, and 5HTT	Dopamine Receptors D2, D3 & D4, Dopamine Active Transporter, Catecholamine Methyl Transferase, Opioid Receptor Mu 1, Serotonin Transporter Gene	(62, 66, 148, 149)
Gambling disorder	OPRM1	Opioid Receptor Mu 1	(150)
Alcohol Use Disorder (In the presence of psychosocial risk factors); Substance Use Disorders, Psychiatric Issues	MAOA	Monoamine Oxidase-A Enzyme	(151–153)
Heroin Dependence	GABRB3	GABA Receptor B3	(154)

These genes have been explored as predictors of bariatric surgery outcomes by Thanos et al. (88, 105).

## 5 Preclinical models of bariatric surgery

Several prior studies have demonstrated a causal relationship between the post-operative RYGB state and the increased risk of a variety of impulsive and compulsive behaviors. Patients who undergo RYGB often experience quicker onset, longer-lasting, and higher blood alcohol concentrations (67–69). Thanos et al. studied this phenomenon using male obese rats that underwent either RYGB or SHAM procedures (70). Both RYGB and SHAM rats were given a choice between water and varying ethanol concentrations over 32 days to assess alcohol consumption. The study found that RYGB rats consumed significantly more alcohol than obese SHAM rats. These data support that obesity is associated with hypodopaminergic signaling by way of reduced D2 and this reward deficiency in the presence of reduced food intake due to the surgery can increase risk for increased alcohol intake (70).

Polston et al. investigated whether RYGB could directly increase alcohol consumption, independent of changes in alcohol absorption and bioavailability (71). They used male obese rats which then underwent either RYGB or SHAM procedures. Both groups were trained to self-administer alcohol with RYGB rats showing greater alcohol intake and greater reinforcement of this behavior compared to SHAM rats. RYGB rats also demonstrated a greater number of responses to self-administer alcohol. These findings thus suggest that RYGB may alter brain reward pathways, increasing reward-seeking behavior rather than affecting the gastrointestinal absorption of alcohol (71).

Ghrelin, a peptide hormone produced in the stomach, is known to increase food intake in the fasting state (72). Hajnal et al. investigated how RYGB affects alcohol consumption in rats and whether changes in ghrelin activity contribute to this effect. In their study, obese male rats underwent either RYGB or SHAM procedures, and then trained to self-administer alcohol. RYGB rats showed significantly increased alcohol-seeking behavior at various ethanol concentrations compared to SHAM controls, and the ghrelin antagonist D-[Lys3]-GHRP-6 reduced ethanol intake in RYGB rats. The results suggest that RYGB may enhance sensitivity to ghrelin regulation and warrant further exploration into how ghrelin could be targeted for treating alcohol abuse in RYGB patients (72). Uchida et al. studied the impact of RYGB on ghrelin levels in obese mice (73). Both SHAM mice and control mice showed lower ghrelin levels, while RYGB obese mice had increased levels. These findings suggest a potential link between RYGB, altered ghrelin signaling, and behavioral responses, as supported by Hajnal et al. (72), who found that RYGB mice with a ghrelin antagonist showed reduced drug-seeking behavior compared to SHAM mice, indicating a complex relationship between ghrelin and reward systems (73). Orellana et al. compared the effects of vertical sleeve gastrectomy (VSG) and RYGB on ethanol intake using male rats and mice with diet-induced obesity (74), with results suggesting that the removal of ghrelin-producing cells in VSG might contribute to reduced ethanol consumption following bariatric surgery (74).

The endogenous opioid system of the body also plays a major role in regulating risk and reward behavior. McGregor et al. focused on whether RYGB affects the mu-opioid receptors of the brain and in turn, increase the risk of addictive behaviors (75). Male obese rats undergoing RYGB had decreased mu-opioid receptor levels in the central amygdala, a region known to regulate stress and anxiety response, which in turn can influence the risk of developing a substance use disorder (75).

Gamma-aminobutyric Acid (GABA) is a key inhibitory neurotransmitter implicated in alcoholism, with increased GABA-A receptors observed in post-mortem brains of individuals with alcohol use disorder due to chronic alcohol reducing natural GABA production (76, 77). It is also well known that increases in GABA-A receptors, especially in the mesolimibic circuitry of the brain, may occur by inhibiting dopamine release at the Nucleus Accumbens (78). McGregor et al. examined changes in GABA-A receptor expression in response to RYGB in male obese rats. RYGB rats exhibited increased [3H] flunitrazepam binding, a marker for GABA-A receptors, in the ectorhinal cortex and primary somatosensory cortex compared to controls. These findings suggest that RYGB surgery leads to overexpression of GABA-A receptors in specific brain regions. It is a possibility that this may contribute to a higher risk of alcohol abuse following the procedure (79).

Hamilton et al. also investigated how RYGB affects the mesolimbic dopamine (DA) system in rats, focusing on its role in eating and addictive behaviors (80). Male obese rats underwent either SHAM or RYGB surgery. After 9 additional weeks, rats were assessed for DA Type 1-like receptors (D1R), Type 2-like receptors (D2R), and DA Transporter (DAT) expression. It was found that SHAM obese rats showed reduced D1R and D2R expression and decreased DAT binding. RYGB rats, showed weight reductions but did not show significant differences in DA receptor expression compared to control rats, suggesting that RYGB may counteract the adverse effects of obesity on the dopaminergic signaling (80). These results were supported by subsequent studies (81, 82).

Thanos et al. tested the effects of RYGB on perception and anticipation of highly palatable foods compared to regular diets in obese male rats (83). Rats first underwent RYGB procedure, or a SHAM procedure. After 3 weeks post-surgery, all rats were conditioned to bacon and chow in a three-chamber Conditioned Placed Preference (CPP) Apparatus. All rats were then scanned twice throughout the duration of the experiment using *in-vivo* positron emission tomography (PET) to measure brain-glucose metabolism (BGluM). Results showed that Bacon CPP was only significant in RYGB rats that had stable weight loss post-procedure. Furthermore, PET of RYGB rats showcased increased BGluM in the regions of the right and midline cerebellum that are involved in subjective processes related to reward and expectation. The data suggests that anticipation of palatable foods in RYGB rats led to activation in the medial parabrachial nuclei, which is significant for gustatory processing, as well as the dorsomedial tegmental area, which is a region known to control reward, motivation, addiction, and cognition. On the other hand, bacon anticipation in control rats showed activation in the retrosplenial cortex and primary visual cortex. Thus, RYGB can lead to alterations in brain activity that influence reward expectations and sensory processing when there is anticipation of intake of palatable fatty foods (83).

Sleeve gastrectome (SG) has also been utilized in preclinical studies. Ding et al. found that compared with the sham operation group, SG rats showed improvements in a number of measures (84). These included improved metabolic and body weight measures, cognitive functions measured by Morris water maze and Y maze, and changes in the hippocampus related to cognitive decline including inhibition of hippocampal apoptosis and decreased phosphorylation of Tau at Ser 404 and Ser396 sites (84). Additional data suggests metabolism is improved in rats after SG, where improvements in insulin sensitivity was observed in both obesity prone and non-obesity probe rats (85). This study also observed decreased body weight, food intake, increased rectal temperature and upregulated brown adipose tissue Ucp-1 protein expression levels (85). Cardiovascular improvements, as well as reductions in bodyweight and increases in excess weight loss, have also been observed in SG treated rats (86). Multiple studies show how compared to RYGB and sham controls, rats who underwent SG consumed less alcohol (74, 87).

A summary of the various behavioral and neurochemical effects of bariatric surgery from preclinical studies is summarized in Table 4.

**Table 4:** Summary effects of bariatric surgery on behavior and neurochemistry in preclinical studies.

**Effect of RYGB compared to Controls**	**Reference**
Increase in alcohol consumption (licking)	(70)
Increase in alcohol self-administration	(71)
Increase in alcohol consumption (licking) Decrease in alcohol consumption with D-[Lys3]-GHRP-6	(72)
Decrease in DAMGO binding, central Amygdala	(75)
Increase in [3H]- Flunitrazepam binding in the ectorhinal cortex	(79)
Normalization of dopaminergic system	(80)
Decrease in [3H]-PK11195 (inflammation)	(155)
Increases in BGluM in midline cerebellum, medial parabrachial nuclei, dorsomedial tegmental area	(83)
Increase in grehlin post RYGB in male mice model	(73)
Decrease in self administration post VSG compared to RYGB	(74)
Increase in dopamine in nigrostriatal pathway	(81)
**Effect of LSG compared to controls**	**Reference**
Improvements in bodyweight and metabolism, improved cognition, decreases in hippocampal apoptosis and phosphorylation of tau	(84)
Improvements in insulin sensitivity, decreased body weight and food intake, increased rectal temperature, upregulated brown adipose tissue Ucp-1 protein expression	(85)
Cardiovascular improvements, reduced bodyweight increase in %EWL	(86)
Decreased alcohol sonsumption compared to controls and RYGB	(74, 87)

## 6 Genetics as a predictor of bariatric surgery outcomes

Various studies have been conducted to investigate the role of genetics in bariatric surgery outcomes. Though the relationship between obesity and However, the genetics associated with addiction risk have been underexplored in this domain. Thanos' group conducted exploratory research on this topic and evaluated GARS and psychosocial data as a means of predicting bariatric surgery outcomes at various time points post-surgery (88). Thirty four patients scheduled to undergo bariatric surgery underwent genetic testing using the GARS assay to evaluate for the presence of the 11 polymorphisms associated with motivation and reward using PCR amplification (43). Patients also submitted presurgical psychological data to evaluate nutritional habits, food addiction, binge eating disorder symptoms, chronic stress and life quality, and sleep. 6-month after the operation, several correlations were identified between various psychosocial questionnaire scores, weight change, and individual risk alleles (88).

The most prevalent homozygote alleles within this study were of the DRD2 and MAO genes detected among 38% and 47% of subjects, respectively. The GARS assay also revealed that 76% of participants fell into the high-risk category for alcoholism (score of 7 or greater). In response to psychosocial questionnaires, many subjects revealed symptoms of depression, trouble with sleep, as well as food cravings.

It was found that the DRD4 risk allele showed significant correlation with change in weight and change in BMI. Receptors for D4 are in several brain regions, with a range of functions such as regulating attention, decision making, reinforcing properties of food, and inhibitory control (89, 90). Scores from the Difficulties in Emotional Regulation Scale (DERS) (91) were positively correlated with the OPRM1 allele. This gene (G allele) has been associated with increased mood disturbances as well as decreased emotional regulation which may result in an increased sensitivity to stressors (92–94). While zero patients in this study were homozygous carriers of the A11G polymorphism, those who were heterozygous for the G allele did display these associated symptoms. DERS results were also positively correlated with pre- and post-surgery BMI, suggesting levels of emotional regulation may have significant correlation with obesity within an individual (95–97). A significant negative correlation was found between the COMT allele, the Eating Expectancies Inventory questionnaire (EEI) (98–100), and the Pittsburgh Sleep Quality Index (PSQI) (101–103). The presence of the COMT risk allele may play a role in the impulsivity that influences binge eating disorder (104).

Thanos et al. also conducted a follow-up with the same group of participants 1 year after bariatric surgery (105). 1-year BMI of subjects revealed a significant negative correlation with the OPRM1 allele and DRD2 alleles. DRD2 was also positively correlated with change in weight and positively correlated with %EWL. ANOVA discovered a significant difference in change of BMI between expressions of the MAOA risk allele. GARS scores were found to be correlated with %EWL, change in weight, and change in BMI. Finally, FCQ scores were revealed to have negative correlation with %EWL and 1-year post-surgical weight loss.

The A1 allelic presence has long been associated with different forms of obesity, including parental/hereditary obesity (106). D2 striatal receptor availability can decrease with obesity and overeating (107–110). The listed findings suggest that surgery may alter D2 sensitivity/activity and the associated reward mechanisms. Additionally, there is evidence supporting an upregulation of D2 receptors after bariatric surgery (80, 107, 111–113). Preclinical research from Thanos et al. showed how obese rats displays reduced D2Rs (158), and how these can be regulated via bariatric surgery (80). In a clinical study, women with obesity showed decreased baseline striatal D2 and D3 expression, which increased with improved body weight 2 years after surgery (113).

The mu-Opioid Receptor is known to modulate reward processing, motivation, and hedonic behaviors (114). Expressions of this receptor have been negatively related to obesity and food cravings (115–118) OPRM1 cerebral availability has been inversely related to external eating behaviors (116). Additionally, when compared to controls, 13 women with obesity showed significantly decreased availability of OPRM1 in the ventral striatum, insula, and thalamus detected with [11(C)]carfentanil PET scans (118).

The MAOA gene encodes for enzymes that breaking down monoamines (119, 120). Polymorphisms of this gene can effect dopamine levels (121). This gene has been correlated with facets of obesity such as weight, BMI, and body fat in Portuguese men (122) as well as female Caucasian twins (120).

A summary of the post-operative results, including bodyweight and genetic data, can be viewed in Table 5.

**Table 5:** Summarizing the bodyweight and psychosocial results of patient data 6-month and 1-year after bariatric surgery.

**6-month**	**DRD4**	**COMT**	**GABR3**
	° Greater change in weight ° Greater change in BMI	° Higher EEI scores	° Higher EEI scores
**1-year**	**DRD2**	**OPRM1**	**GARS**
	° Lower BMI ° Greater change in weight	Higher %EWL ° Greater change in weight ° Greater change in BMI	° Lower BMI

Results suggesting that those of a higher genetic susceptibility to impulsive/compulsive behavior are more receptive to weight-loss surgery.

## 7 Psychosocial risk factors related to epigenetics in predicting bariatric surgery outcomes

The following psychosocial questionnaires have been utilized to measure obesity-related behaviors. The Eating Attitudes Test-26 (EAT-26), Food Cravings Questionnaire-Trait Reduced (FCQ-TR) and Eating Expectancies Inventory (EEI) questionnaires serve as a measure of nutritional habits (98–100). In one study, 262 women completed the EEI before undergoing bariatric surgery. The results of this study suggest that the EEI is a reliable and valid indicator of eating patterns/pathology and postsurgical weight loss outcomes (123). Additionally, the modified Yale Food Addiction Scale 2.0 (mYFAS 2.0) assesses food addiction (124). This questionnaire has been used as a measure of cravings in bariatric surgery patients. Similarly, the Weight Influenced Self-Esteem Questionnaire (WISE-Q) measures binge eating disorder symptoms (125). The DERS and Center for Epidemiologic Studies Depression Scale (CESDS) questionnaires assess anxiety/depression within participants (91, 126). One study utilized the DERS questionnaire among both obese and control adult female participants. This study revealed that DERS scores were higher in the obese group which is likely due to individuals with higher BMI exhibiting less effective inhibition in the amygdala during reappraisal. This alteration likely contributes to an increased difficulty in regulating emotions in obese women (127). Finally, chronic stress and life quality and sleep can be measured through the Chronic Stress Index (CSI) and Pittsburgh Sleep Quality Index (PSQI), respectively (101–103). A cross-sectional study measuring the association between sleep quality and obesity in Korean adults was completed which utilized the PSQI, associating obesity with insufficient sleep in women (160). A meta-analysis revealed that shorter sleep duration is significantly associated with obesity (128). Administering these questionnaires will help in aiding psychosocial concerns following bariatric surgery that have been seen to be linked to these various sociodemographic factors.

## 8 Bariatric surgery: implications for personalized medicine

Here we have detailed some of the neurogenetic and molecular mechanisms that may be associated with genotypical receptivity to bariatric surgery. Results from Thanos et al., generally detail which genotypes are more receptive to treatment based on their individual GARS scores (88, 105). Further studies could target nutrigenomics, correlating this concept with post-surgery weight loss. Nutrigenomics, specific nutrition plans targeting genotypical deficiencies, is an important factor for personalized medicine (129). This concept has been explored by Blum's group and others especially in the realms of targeted nutritional replacement therapies, notably amino acid therapies, for addiction treatment and induction of dopamine homeostasis (130).

A personalized approach can be important for patient education. Genetic counseling can lead to lifestyle changes that promote health and wellbeing (131). The genotypes discussed in this review typically deal with psychiatric neurogenetics. Other investigators in the field of obesity genetics have delved into the genetics of metabolic networks, adiposity, and hormone receptors (132–134).

Adiposity genetics was observed as a predictor of post-bariatric surgery weight loss. In this study, 13 SNPs related to adiposity were associated with post bariatric weight loss (132). These included SNPs in the following genes: *PKHD1*, ST8SIA2/SLCO3A1, *PRKD1, NUP54/SCARB2, GBE1, AGBL4, BCDIN3D, NLRC3, TCF7L2, BCL2, MEIS1, RSPO3, GDF5, CCDC92, DNM3-PIGC*. Patients genetically predisposed to low body mass index had lower weight loss after bariatric surgery. In 2013, two genome-wide association studies (GWASs) exploring weight reduction following bariatric surgery, each comprising 1,143 and 1,018 participants, respectively, identified significant associations between surgery-induced weight loss and the SNPs rs728996 (located in PKHD1) and rs17702901 (found in ST8SIA2) (135, 136).

Measures such as genetic considerations provide a framework for implementing personalized medicine approaches in clinics such as Bariatric Surgery centers. This suggests a more intricate level of tailoring, encompassing diagnostic, screening, treatment, and management strategies rooted in genomics and other pertinent factors, along with a methodical integration of this personalized approach into healthcare delivery (137). The benefit of a test like GARS is that it can pinpoint individuals at elevated risk and offering tailored interventions to mitigate risks, thus preempting the onset of disease symptoms (137–139). Genetic testing in personalized and precision medicine encompasses a wide range of disorders, including schizophrenia, bipolar disorder, posttraumatic stress disorder, cardiac disease, metabolic disease, renal disease, and substance abuse (137, 140) and among specific sub populations such as veterans (140).

## 9 Conclusion

The obesity epidemic has become a global issue, impacting more than one billion people worldwide. This public health problem is often described as being caused by various genetic, psychosocial factors. One of the most effective strategies for treating morbid obesity and achieving significant weight loss is bariatric surgery. Recent focus on precision medicine approaches have expanded into bariatric surgery in an effort to better understand and achieve improved outcomes and reduce risk for post-operative weight regain and addiction transfers. Addiction transfers, including substance and non-substance addictions, are well established concerns for post-bariatric patients. Genetic data, psychological data, and other data (156, 157) can be utilized to guide and inform clinical decisions in bariatric surgery.
